# Jumping in lantern bugs (Hemiptera, Fulgoridae)

**DOI:** 10.1242/jeb.243361

**Published:** 2021-12-09

**Authors:** M. Burrows, A. Ghosh, G. P. Sutton, H. M. Yeshwanth, S. M. Rogers, S. P. Sane

**Affiliations:** 1 National Centre for Biological Sciences, Tata Institute of Fundamental Research, GKVK Campus, Bellary Road, Bengaluru 560 065, India; 2 Department of Zoology, University of Cambridge, Cambridge CB2 3EJ, UK; 3 School of Life Sciences, University of Lincoln, Brayford Pool, Lincoln LN6 7TS, UK; 4 Department of Entomology, University of Agricultural Sciences, GKVK (Gandhi Krishi Vigyan Kendra), Bengaluru 560 065, India

**Keywords:** Locomotion, Take-off, High-speed imaging, Escape movements

## Abstract

Lantern bugs are amongst the largest of the jumping hemipteran bugs, with body lengths reaching 44 mm and masses reaching 0.7 g. They are up to 600 times heavier than smaller hemipterans that jump powerfully using catapult mechanisms to store energy. Does a similar mechanism also propel jumping in these much larger insects? The jumping performance of two species of lantern bugs (Hemiptera, Auchenorrhyncha, family Fulgoridae) from India and Malaysia was therefore analysed from high-speed videos. The kinematics showed that jumps were propelled by rapid and synchronous movements of both hind legs, with their trochantera moving first. The hind legs were 20–40% longer than the front legs, which was attributable to longer tibiae. It took 5–6 ms to accelerate to take-off velocities reaching 4.65 m s^−1^ in the best jumps by female *Kalidasa lanata*. During these jumps, adults experienced an acceleration of 77 ***g***, required an energy expenditure of 4800 μJ and a power output of 900 mW, and exerted a force of 400 mN. The required power output of the thoracic jumping muscles was 21,000 W kg^−1^, 40 times greater than the maximum active contractile limit of muscle. Such a jumping performance therefore required a power amplification mechanism with energy storage in advance of the movement, as in their smaller relatives. These large lantern bugs are near isometrically scaled-up versions of their smaller relatives, still achieve comparable, if not higher, take-off velocities, and outperform other large jumping insects such as grasshoppers.

## Abstract (precis)

**Summary:** Lantern bugs are large insects that jump at high-take-off velocities using a catapult mechanism that matches the performance of their much smaller planthopper relatives

## INTRODUCTION

Jumping has evolved many times across different groups of insects as a rapid and effective form of locomotion serving various actions in natural behaviour. Some of the most effective exponents of jumping, in terms of take-off velocities achieved, are found in the Auchenorrhyncha, a diverse group of hemipteran bugs containing the froghoppers (Cercopoidea), leafhoppers (Cicadellidae), treehoppers (Membracidae) and planthoppers (Fulgoroidea). Their jumping prowess is illustrated by the froghopper *Philaenus spumarius* (Cercopoidea, family Aphrophoridae) (Burrows, 2003) and the planthopper *Issus coleoptratus* (Fulgoroidea, family Issidae), which generate among the fastest take-off velocities recorded for insects (Burrows, 2009a,b). This jumping ability, which is propelled by the hind legs, is a defining feature of the Auchenorrhyncha (Kristensen, 1975).

Muscles generate the most mechanical energy when they contract slowly (Zajac, 1989), and this poses a problem for small animals that must produce rapid forceful movements, such as those needed for jumping, as they cannot exploit the leverage provided by long legs and have only a short time available for acceleration before the legs are completely extended. In small jumping insects, therefore, catapult mechanisms are used, which decouple muscle contractions from the eventual rapid movement (Bennet-Clark, 1975; Burrows, 2003; Sutton et al., 2019). This is achieved by slow muscle contractions distorting mechanical specializations of the cuticular exoskeleton, which act as elastic energy stores, whilst the propulsive legs do not move. Sudden recoil of these energy stores releases the stored energy and powers the rapid movements of the legs, accelerating the body more quickly and with greater power than could be delivered by the direct action of muscle alone. The recoil-driven jumps of insects are thus a subset of ‘latch-mediated spring-actuated’ behaviours, which are not directly actuated by muscle contraction, but instead by the quick recoil of an elastic energy store (Ilton et al., 2018; Longo et al., 2019).

**Fig. 1.**
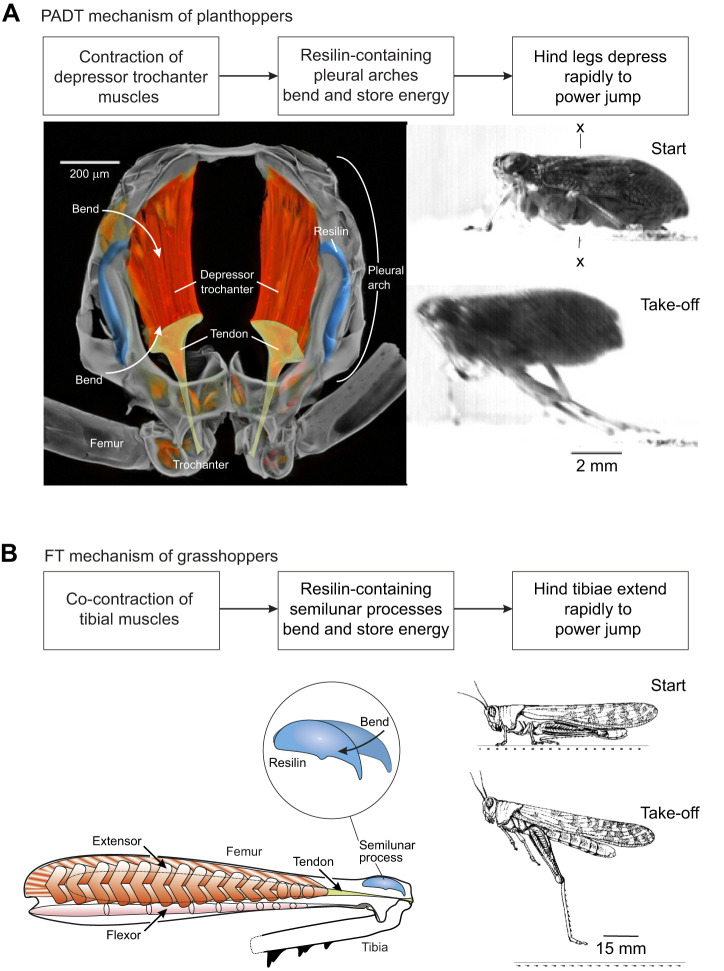
**Diagrammatic representation of the recoil-driven jumping mechanism in two groups of insects.** (A) The pleural arch and depressor trochanter (PADT) mechanism in hemipteran planthoppers illustrated by *Issus coleoptratus*. Left: cross-section through the metathorax at the level shown by x in the photograph to the right, with structures involved in jumping labelled (modified from Siwanowicz and Burrows, 2017). Right: *I. coeloptratus* shown prior to and at take-off. (B) The hind leg femoro-tibial joint (FT) mechanism in orthopteran grasshoppers, illustrated by *Schistocerca gregaria*. Left: diagram of a hind leg of a grasshopper showing the principal structures involved in jumping, with distortion of the energy-storing semilunar process shown as an inset (modified from Rogers et al., 2016). Right: illustrations of a locust prior to and immediately after take-off.

In the Auchenorrhyncha, the metathoracic pleural arch, a complex bow-like thickening of the body wall, acts as the principal elastic energy store; it is slowly compressed during contraction of the massive depressor trochanter muscles of the hind legs before the jump occurs. These two elements form the PADT (pleural arch+depressor trochanter) mechanism (Fig. 1A). Together, the pleural arches and depressor trochanter muscles occupy much of the space within the metathorax (Burrows et al., 2008; Siwanowicz and Burrows, 2017). In common with the energy storage structures of many other jumping insects, the pleural arches are associated with the elastic protein resilin, which allows the efficient return of the stored energy, the rapid restitution of the original body shape, and prevents damage (Burrows et al., 2008).

All the species in the Auchenorrhyncha that have been shown to be capable of jumping with take-off velocities of more than 3 m s^−1^ have been small (most <40 mg). Similarly high take-off velocities have been found in larger insects (>100 mg), such as grasshoppers and locusts, but these all belong to the Orthoptera (Caelifera) and use a jumping mechanism based on the storage of energy in, and rapid extension of, the hind femoro-tibial joint (FT mechanism) (Fig. 1B). In the 10–40 mg size range dominated by Auchenorrhyncha, there are only a few fast-jumping Orthoptera, such as the pygmy mole cricket *Xya capensis* (Burrows and Picker, 2010). This pattern suggests that there may be limitations on the performance of PADT and FT jumping mechanisms based on size. We were therefore fortunate to obtain specimens of lantern bugs (Fulgoroidea, family Fulgoridae), which have masses 20–600× heavier than previously studied planthoppers (e.g. Burrows et al., 2019) or froghoppers (Burrows, 2006), in which for the first time we can compare the performance of large insects with a PADT jumping mechanism with smaller members of the group. The planthoppers or Fulgoroidea (=Fulgoromorpha) are a group of 21 families within the Auchenorrhyncha that share many common morphological features (Cryan and Urban, 2012; Skinner et al., 2020), amongst which is a hind leg-movement synchronising mechanism based on interlocking gears on their hind trochantera as nymphs (Burrows and Sutton, 2013; Siwanowicz and Burrows, 2017). This group is therefore ideal for comparing jumping performance across a size range of three orders of magnitude (1.65–530 mg). This paper asks whether a catapult mechanism alone can provide the propulsion for jumping in these large lantern bugs? Alternatively, does jumping in these large bugs need to be supplemented by other mechanisms, such as wing movements, or disproportionately long legs? We show that these large hemipterans are near isometrically scaled-up versions of their smaller relatives and that despite their large mass, they can achieve equivalent take-off velocities when jumping, which surpass the performance of other large jumping insects such as locusts.

## MATERIALS AND METHODS

### Insects

Eight live adult males and females of the lantern bug, *Kalidasa lanata* (Drury 1773), were collected in October 2019 from the rain forest in Agumbe, Karnataka, India (the second wettest place in India), and were videoed the next day in Bengaluru. The species was originally named *Cicada lanata* but the genus was changed to *Kalidasa*, by Kirkcaldy in 1900, after the most celebrated Sanskrit poet, who is believed to have lived during the 4th to 5th century bce. Two live specimens of *Pyrops candelaria* (Linnaeus 1758) (see also Kershaw and Kirkaldy, 1910) were also collected from lowland forest around Danum, Sabah, Malaysia, and were videoed on the same day in 2004. A single preserved specimen of an unknown species of *Pyrops* from Dong Nai, Vietnam, was kindly given to M.B. by Roy Bateman. *Purops candelaria* exemplifies why this group are called lantern bugs: there was an erroneous belief that the tapering 5 mm long snout projecting forward from the head emitted light. By contrast, adult *K. lanata* have no snout, but instead have a single 0.5 mm thick process that projects backwards for approximately 4 mm from the top of the head. The true function of these protrusions is unknown.

### Anatomy

After the kinematics of jumping were recorded, the external morphology of the legs was analysed in all live insects. Additional analyses were made subsequently when they were preserved in 70% alcohol. Leg and body lengths of *K. lanata* were measured to an accuracy of 0.1 mm from images captured on a Nikon SMZ 25 microscope with a Nikon DS-Ri2 camera (Nikon Instruments, Melville, NY, USA) (Table 1). Body masses were determined to an accuracy of 0.1 mg with a BSA224S-CW balance (Sartorius Lab Instruments, Gottingen, Germany). *Pyrops candelaria* was measured to the same accuracy in Borneo with an unrecorded make of microscope and balance.

**Table 1.**
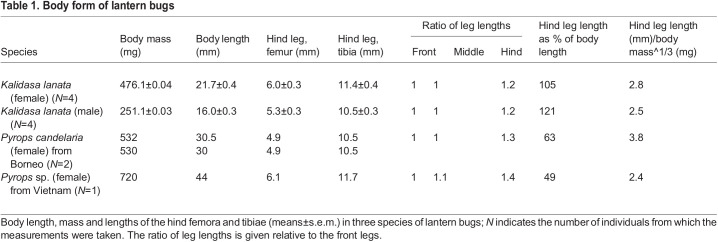
Body form of lantern bugs

### Kinematics

Jumps by *K. lanata* were recorded in a chamber with an internal width of 150 mm, height of 90 mm and depth of 30 mm. Images were captured at a rate of 5000 s^−1^ with an exposure time of 0.06 ms and a resolution of 1280×800 pixels with a single Phantom VEO640L camera (Vision Research, Wayne, NJ, USA) fitted with a 105 mm Nikon lens. Jumps of *P. candelaria*, were recorded in a chamber with an internal width of 240 mm, height of 90 mm and depth of 50 mm, at the same frame rate with a single Photron Fastcam SA3 camera (Photron, High Wycombe, UK) with an exposure of 0.05 ms. All insects were recorded in their respective chambers under the same lighting. Images from a camera were saved directly to a computer for later analysis. The insects were free to move in chambers made of 1.9 mm thick glass with the floor, sidewalls and ceiling internally lined with 12 mm thick, closed-cell foam (Plastazote, Watkins and Doncaster, Leominster, UK). Only jumps from the floor were analysed for jump performance so that the effects of gravity were the same for all measurements. Some jumps were made from the front glass wall and gave views of leg movements as seen from underneath the body, but information about performance was not extracted or used because of leg slippage. Most jumps occurred spontaneously and without any overt mechanical or other stimulus. Different jumps, viewed either from the side or from the front, are illustrated. Some jumps occurred when a 100 µm silver wire or a fine paint brush was brought close to the insect but before they made contact with it. No differences in the kinematics of a jump were seen in these two situations. The camera pointed at the centre of the chamber and most jumps were in the image plane of the camera. Those that deviated to either side of this plane by more than ±30 deg were not included in the analysis (at 30 deg there is a 13% length measurement error). Tracks of the movements of particular joints of the legs and of the body were made manually frame by frame with Tracker software (http://physlets.org/tracker/). Take-off was indicated when the hind legs lost contact with the ground, and was designated as time *t*=0 ms. The acceleration time for take-off was defined as the period from the first detectable propulsive movement of the hind legs until take-off when the hind tarsi lost contact with the ground. A point on the body that could be recognized in successive frames and which was close to the centre of mass (measured approximately by balancing dead insects on the point of a pin) was selected for measurements of the trajectory. The angle subtended by a line joining these points after take-off, relative to the natural horizon, gave the trajectory angle. The body angle at take-off was defined as the angle subtended by the longitudinal axis of the insect relative to the natural horizon. The results are based on the analysis in Bengaluru in 2019 of high-speed videos of 30 jumps by four female *K. lanata* with a minimum of three jumps by each individual at a temperature of 25–30°C. The four live males showed no willingness to jump. Two jumps by each of two female *P. candelaria* were recorded in Sabah, Borneo, in 2004 at the same range of temperatures. Measurements are given as means±s.e.m. for an individual and as mean of means (grand means) for members of one species. Data for comparative and scaling studies came from previously published and unpublished measurements of morphometry and jumping performance (see details in Table 3), with each point representing the best jumping performance or average dimensions of a different species. Scaling relationships between body mass, leg lengths and jumping performances were analysed using standardised major axis regression, which takes into account errors on both *x*- and *y*-axes (Warton et al., 2012). Statistical tests and analyses were performed using R (https://www.r-project.org/) and the standardised major axis regression package SMATR. Tests were made of the significance of fitted regressions (difference from slope=0) and whether the slopes differed from isometric scaling. Significance levels were set at α=0.05.

## RESULTS

### Shape of body and legs

**Fig. 2.**
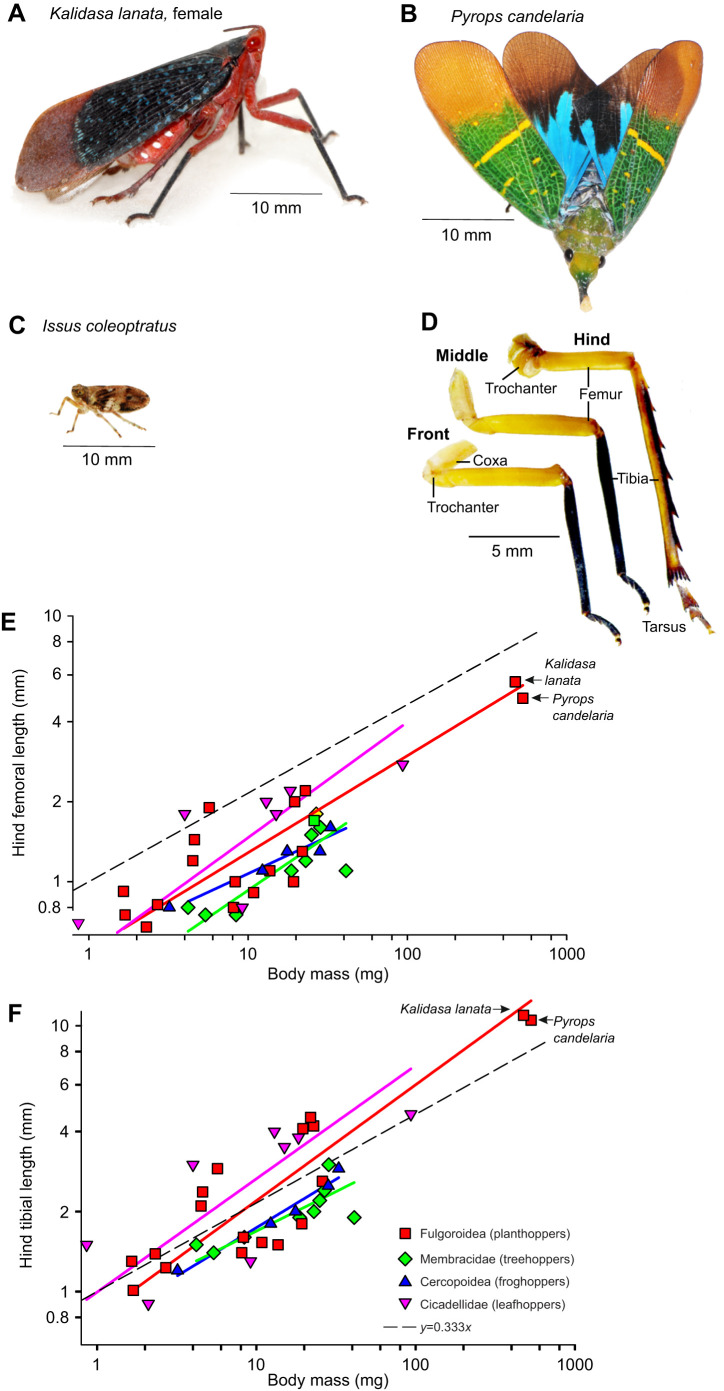
**Form of the body and legs of lantern bugs.** (A) Photograph of an adult female *Kalidasa lanata* viewed from the side. (B) Photograph of *Pyrops candelaria* viewed dorsally and with the wings partly open. (C) Photograph of the planthopper *Issus coleoptratus* viewed from the side for comparison of size. (D) Photograph of the three left legs of *Pyrops* sp. viewed from their lateral surfaces. (E,F) Log-log plots of the mass of 40 species of auchenorrhyncan jumping insects in different (super)families plotted against the lengths of their hind femora (E) and tibiae (F). The dashed lines have a slope of 0.33. For the hind femur, none of the lines fitted to each family grouping had a slope significantly different from 0.33. For the hind tibia, fitted lines did not differ significantly from 0.33 except for the Fulgoroidea, where the slope was 0.43.

The two species of lantern bugs used here were both brightly coloured. *Kalidasa lanata* had a red body, dark marking on the wings with translucent patches tinged with blue, and a red abdomen with white spots on its lateral surfaces (Fig. 2A). *Pyrops candelaria* had green and orange front wings and blue patches on the dorsal surface of the hind wings (Fig. 2B). The piercing mouthparts of both species extended ventrally and as far posteriorly as the middle segments of the abdomen. In female *K. lanata*, the terminal abdominal segments were surrounded by numerous densely packed strands of white wax.

Adult female *K. lanata* had a mean body mass of 476.1±0.04 mg and a body length, as measured from the anterior tip of the proboscis to the posterior tip of the abdomen, of 21.7±0.38 mm (*N*=4) (Table 1). The wings extended beyond the tip of the abdomen by approximately 8 mm when they were closed and folded along the length of the body. Male *K. lanata* were smaller, with a body mass of 251.1±0.03 mg (47% less than females) and a body length of 16.0±0.29 mm (26% decrease) (*N*=4) (Table 1). The two females of *P. candelaria* had a mass of 532 and 530 mg and a body length of 30.5 and 30 mm, respectively (as measured from the anterior tip of the proboscis to the posterior tip of the abdomen), and the single specimen of an unidentified *Pyrops* species from Vietnam was even heavier at 720 mg with a body length of 44 mm (Table 1). The huge size of these species compared with other planthoppers is illustrated by a photograph, at the same magnification, of *I. coleoptratus* (family Issidae), which had a body mass of 26 mg and a body length of 7.4 mm (Fig. 2C).

The femora and tibiae of all the legs of both species were of similar tubular shape, with none having an increased volume consistent with containing enlarged tibial extensor muscles (Fig. 2D). Dissections of the thorax showed enlarged trochanteral depressor muscles in the metathorax, as described in other planthoppers (Burrows, 2009a). The coxae of the front and middle legs were elongated and mobile in contrast to those of the hind legs, which were fused to the metathorax, as in other planthoppers (Burrows, 2009a). The femora of each leg of female *K. lanata* were all of similar lengths (front 6.7±0.3 mm, middle 6.0±0.1 mm, hind 6.0±0.3 mm, *N*=4). By contrast, the tibiae of the hind legs were 65% longer than those of the front and middle legs (front 7.0±0.2 mm, middle 6.9±0.1 mm, hind 11.4±0.4 mm) and resulted in a ratio of legs lengths when expressed relative to the front legs of; 1 front: 1 middle: 1.2 hind*.* In *P. candelaria*, this ratio was 1:1:1.3 or 1.4 (Table 1). The hind tibiae alone bore a series of five outwardly pointing spines and an array of shorter spines at their articulation with the hind tarsi. A similar array of short spines was present at the joints in each segment of the tarsi. These shorter spines were pushed into the ground when the hind legs were moved rapidly to propel a jump. In froghoppers (Aphrophoridae), the short spines have been shown to penetrate the surface of leaves from which they jumped and thus increased traction and reduced the likelihood of slippage (Goetzke et al., 2019).

The scaling relationships between the length of a hind femur and tibia were compared across the jumping Auchenorrhyncha (Fig. 2E,F). The graphs show log-log plots of the body masses of 40 species of Auchenorrhyncha plotted against the lengths of their hind femora (Fig. 2E) and tibiae (Fig. 2F) separated by family groupings (Fulgoroidea, Membracidae, Cercopoidea and Cicadellidae). The dashed lines have a slope of 0.33, which is the slope that would be expected from an isometric relationship between body mass and limb length (based on volume scaling to linear measurements). For the hind femora lengths, none of the fitted lines for any of the families had a slope significantly different from 0.33 (range 0.28–0.44; Table 3, Fig. 2E). For the Fulgoroidea specifically, the regression had a slope of 0.37 (significance of fitted regression: *R*^2^=0.77, *P*=2.1×10^−6^, range estimate of slope 0.29–0.48), but as with the other families, the slope did not significantly differ from an isometric expectation of 0.33 (*R*_16_=0.21, *P*=0.403). Both *K. lanata* and *P. candelaria* lay close to the fitted line, indicating that the proportions of their hind femora were isometrically scaled-up versions of their much smaller relatives.

The hind tibiae of the cercopoids, cicadellids and membracids also appeared to follow near-isometric scaling with body mass (fitted slopes 0.36, 0.43 and 0.30, respectively, Fig. 2F; none were significantly different from 0.33, see Table 3), but in the fulgoroids there was evidence for a positive allometric relationship, as the fitted slope was 0.43 (significance of regression, *R*^2^=0.79, *P*=8.9×10^−7^, range estimate of slope 0.34–0.59). This slope was significantly different from 0.33: *R*_16_=0.49, *P*=0.038. The hind tibiae of *K. lanata* and *P. candelaria* were approximately 38% longer than would be predicted from an isometric relationship.

### Kinematics of jumping

All jumps by both *K. lanata* and *P. candelaria* were propelled in the same way by rapid movements of the hind legs. The following description refers to 30 jumps recorded from four female *K. lantana*. The hind legs were rapidly extended but there were no consistent movements of the other legs that indicated a contribution to propulsion. Similarly, the wings remained closed and folded along the body during all recorded take-offs and thus could also not contribute any additional propulsion. They were only opened and moved at variable times after take-off to transition to flapping flight.

**Fig. 3.**
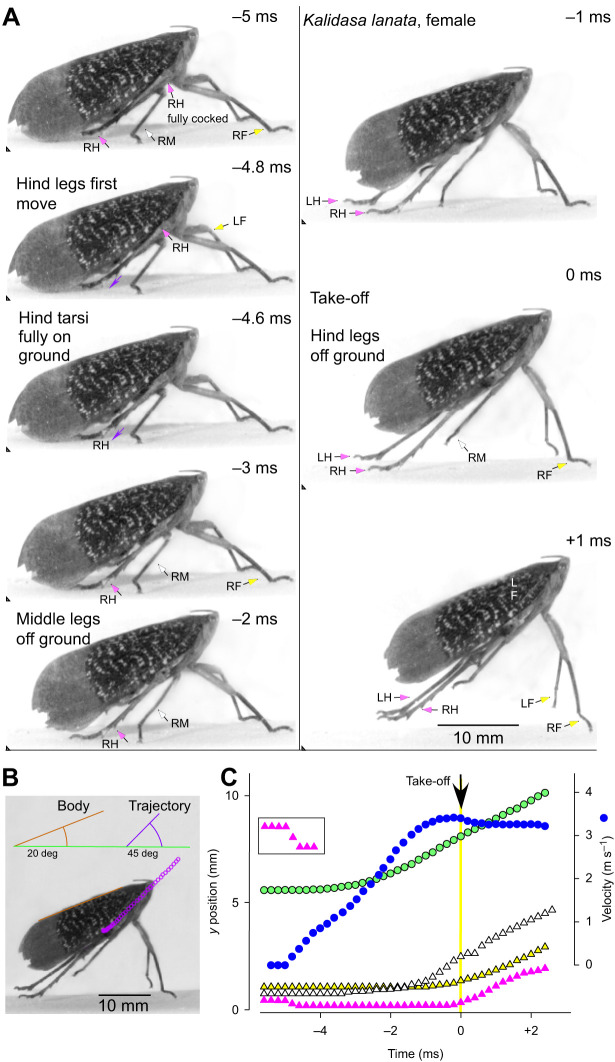
**Jump from the horizontal by a female *K. lanata* viewed from the side.** (A). Images from a high-speed video of a jump were captured at 5000 s^−1^ and with an exposure time of 0.06 ms in this and subsequent figures. The images are arranged in two columns with their timing given relative to take-off at time=0 ms. In this and Figs 4–6, the front legs (LF, left front; RF, right front) are indicated by arrows with yellow heads, the middle legs (LM, left middle; RM, right middle) by arrows with white heads and the hind legs (LH, left hind; RH, right hind) by arrows with pink heads. The triangles in the bottom left hand corners of each image indicate a constant spatial reference point. The thinner purple arrows (frames −4.8 and −4.6 ms) show the initial movements of the right hind tarsus to contact the ground. (B) Image of the body at take-off showing its angle of 20 deg relative to the horizontal and the 43 deg angle of the trajectory (measured from the tracked movements of the thorax once airborne). (C). Plots against time of movements on the *y*-axis of the right front (yellow), middle (white) and hind tarsi (pink triangles), the right eye (green circles) and with the measured velocity of the insect (blue circles, m s^−1^) superimposed. The inset box at higher *y*-axis magnification shows the initial rapid movement of the hind tarsus until it fully contacts the ground and bears the weight of the body.

**Fig. 4.**
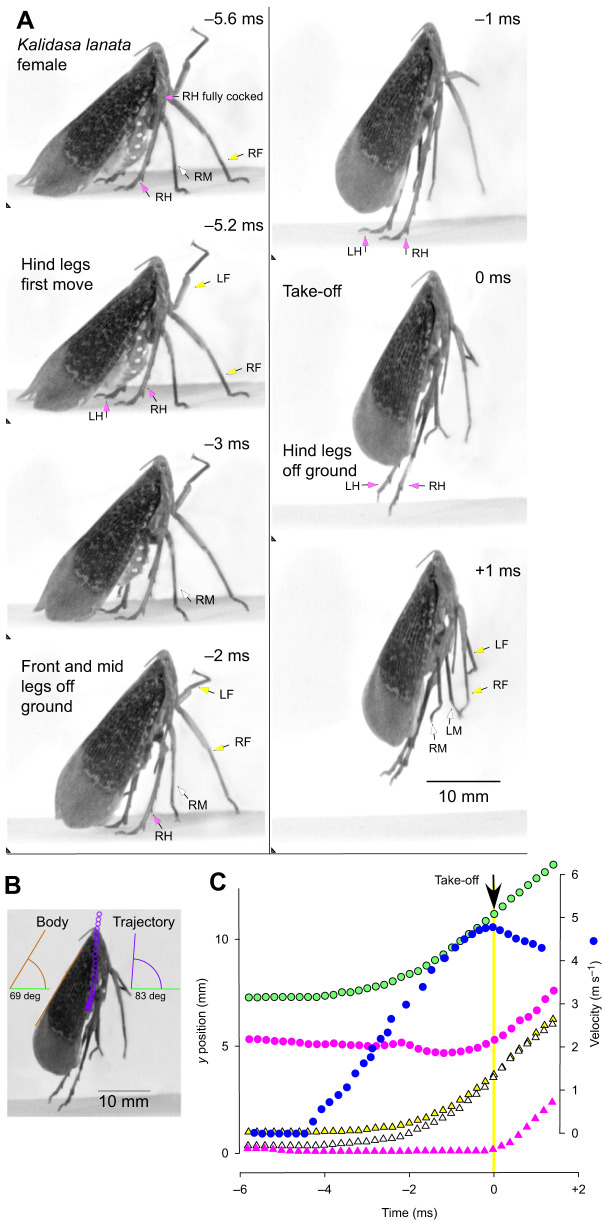
**Jump from the horizontal with a steep trajectory by another female *K. lanata*.** (A) Images of a jump captured at 5000 s^−1^ are arranged in two columns at the times indicated relative to take-off as in Fig. 1. (B) The angle of the body at take-off, relative to the ground was 69 deg and the angle of the trajectory once airborne was a steeper 83 deg. (C) Plots against time of movements on the *y*-axis of the right front (yellow), middle (white) and hind tarsi (pink triangles), the right hind femoro-tibial joint (pink circles), the right eye (green circles) upon which the measured velocity (blue circles, m s^−1^) was superimposed.

**Fig. 5.**
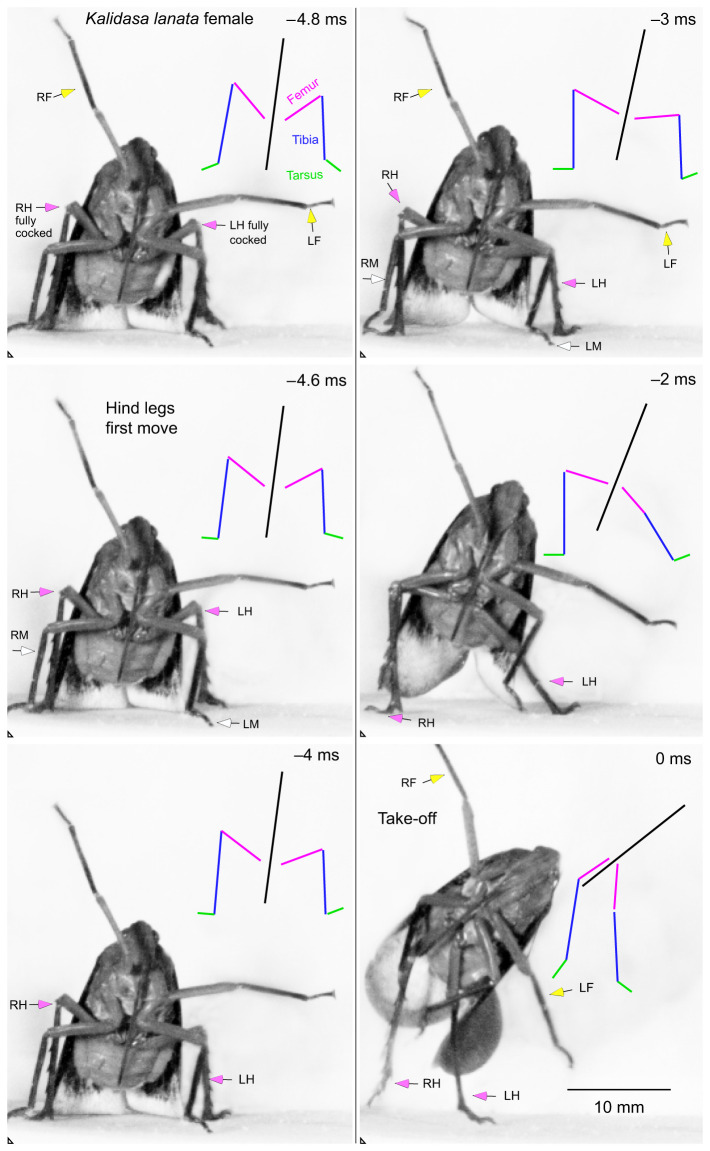
**Jump towards the camera by a female *K. lanata*.** The left and right hind legs initially moved simultaneously at their coxo-trochanteral joints to propel the jump. These movements are also represented in the colour-coded stick diagrams (femur, pink: tibia, blue: tarsus, green: black, longitudinal body axis) that accompany each frame. The femoro-tibial joint of the left hind leg extended earlier and then more fully than the corresponding joint of the right hind leg. The same conventions are also used in Fig. 6. The selected images are arranged in two columns.

**Fig. 6.**
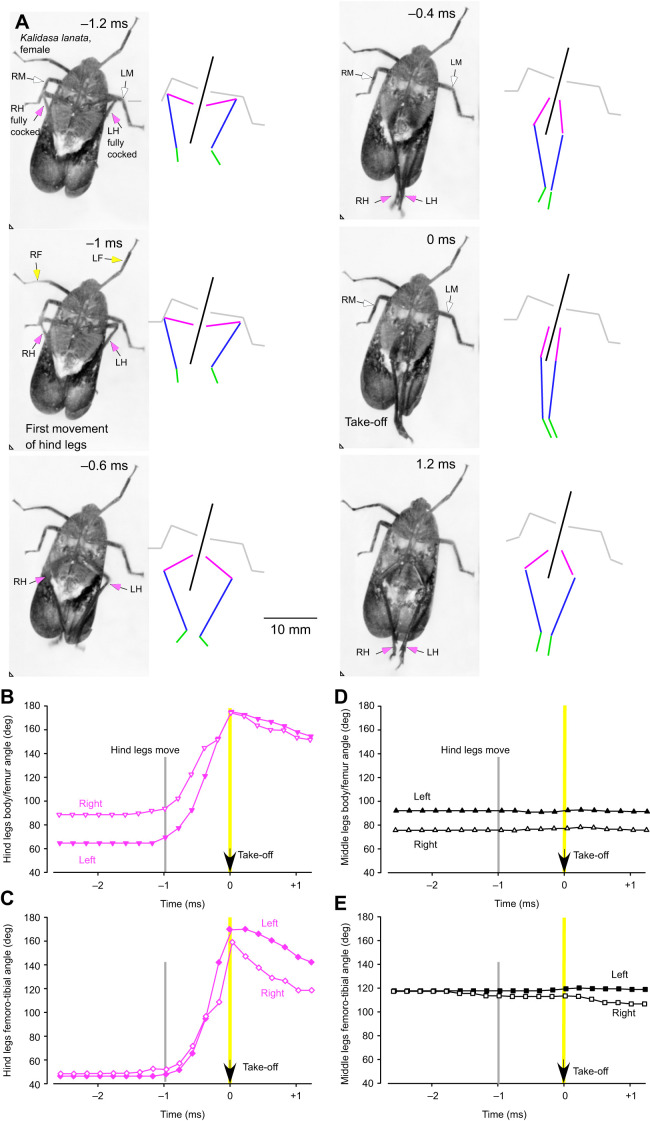
**Rapid hind leg movements in a jump from a vertical glass surface and viewed from underneath.** (A) Selected images at the times indicated are accompanied on their right by stick diagrams of the hind leg joints using the same colour coding as in Fig. 4, with the addition of the middle legs shown in grey. The two hind legs progressively depress and extend but slip on this surface so that their propulsive movements are completed in 1 ms. By contrast the middle legs do not move and thus cannot contribute to propulsion. (B,C) Plots of the angular changes between the body and femora (B), and at the femoro-tibial joints (C) of the right and left hind legs associated with propulsion of the jump. (D,E) These same joints in the left and right middle legs show no changes that correlate with propulsion. The start of movements by the hind legs are indicated by the vertical grey lines, take-off by the yellow lines.

Before a jump was initiated spontaneously, both hind legs were rotated forward at their coxo-trochanteral joints so that the whole femur was also swung forwards with the femoro-tibial joint articulations, as viewed from the side, achieving their most anterior and dorsal (fully cocked) position (Figs 3A and 4A). This initial position of the coxo-trochanteral joints was adopted by both hind legs, as could be seen when the insects were videoed jumping toward the camera or when their legs were viewed from underneath (Figs 5A and 6A). No jumps were observed that were not preceded by this movement.

On some occasions, the levation would be completed but no jump would subsequently follow. Instead, the joints would simply depress slowly so that hind leg movements associated either with walking or adjustments of posture could occur. When a jump was to be generated, the first depression movements of the two hind legs from their fully cocked position were synchronous (within the 0.2 ms intervals set by the video frame rate) and defined the start of the acceleration period of a jump to take-off. The depression of the trochantera were usually accompanied by movement of the femora, but movement of the intervening trochantero-femoral joint meant that the most readily measured angle between the body and the femur would sometimes appear to indicate that the two hind legs were subtending initially different angles even though the coxo-trochanteral angles were the same (see stick diagrams of the hind legs in Figs 5 and 6).

The first movement of the hind coxo-trochanteral joints pushed the hind tarsi into full contact with the substrate along their entire length from an initial position where only the tips were on the ground. This first movement was thus faster than the following depression movements, which had to raise and propel the mass of the body (Fig. 3C). These later movements were also accompanied by extension of the femoro-tibial joints so that the hind legs gradually straightened to reach their full extent at take-off. These propulsive movements of the hind legs had a further consequence in that the front and middle legs were lifted from the substrate in a sequence that depended on the starting angle of the body: if this angle was shallow, the middle legs were the first to lose contact (Fig. 3A,C); if it was steeper, the front legs were first (Fig. 3A,C). The posture of the middle and front legs prior to the jump acceleration strongly influenced the angle of the body relative to the substrate and the trajectory of the jump by setting the angle through which the thrust of the hind legs was delivered. For example, in some jumps, the body angle was shallow (20 deg in Fig. 3B), in others it was steeper (69 deg in Fig. 4B). These angles were reflected in the subsequent trajectory of the jump after take-off (45 and 83 deg, respectively, in these two examples) (Figs 3B and 4B).

The sequence and pattern of the leg movements were also analysed from jumps in which take-off was attempted from the vertical glass front wall of the chamber (Fig. 6). This allowed the movements of both hind legs to be observed at the same time, but the glass surface provided little traction for a jump. As a result, both hind legs slipped and completed their full depression and extension movements in 1 ms (Fig. 6A), or approximately one-fifth of the time it took when propelling the mass of the body from a surface on which it did not slip. At the point of take-off, the two hind tarsi were moving medially and so came into contact with one another, demonstrating the persistence of force in their respective muscles. Again, the middle and front legs made no contribution to these propulsive movements. Tracking movements of the different parts of the legs showed that the hind legs executed a complex path in which they were first depressed about the coxo-trochanteral joint, propelling the body forwards and upwards when viewed from underneath. The following features of the jump were also seen. First, both the body/femur angles (reflecting changes in the coxo-trochanteral angles) occurred synchronously in the left and right hind legs (Fig. 6B,C). Second, progressive increases in the angles of the hind femoro-tibial joints of both hind legs occurred at the same time (Fig. 6D,E). Third, no changes in either of these joint angles happened in the left or right middle legs (Fig. 6B-E).

### Jumping performance

From these kinematic data, the jumping performance of the lantern bugs was calculated (Table 2). The mean acceleration time of a jump was 4.9±0.5 ms in female *K. lanata* and 5.9±0.5 ms in the heavier *P. candelaria*. Female *K. lanata* had a mean take-off velocity of 3.0±0.5 m s^−1^ and achieved a maximum take-off velocity of 4.65 m s^−1^, whereas *P. candelaria* had a mean take-off velocity of 3.1±0.3 m s^−1^ with a maximum velocity of 3.9 m s^−1^.

**Table 2.**
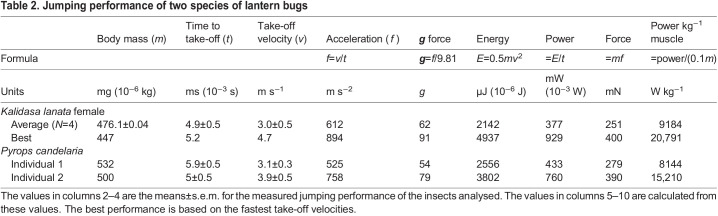
Jumping performance of two species of lantern bugs

These short acceleration times of 4.9–5.9 ms coupled with high take-off velocities resulted in accelerations of 600–900 m s^−2^ with ***g*** forces of 54–91 being experienced by female *K. lanata*. The energy required to move the large mass of these insects to these high take-off velocities approached 5000 µJ and the power exceeded 900 mW in the fastest jumps. Assuming that the muscle propelling the movements of the hind legs represented 10% of body mass, as has been measured for other jumping planthoppers (Burrows, 2006; Burrows and Bräunig, 2010), then the power requirements reached 21,000 W kg^−1^ in the fastest jumps.

## DISCUSSION

Several lines of evidence suggest that the jumps of the large lantern bugs studied here were propelled by a mechanism similar to that used by smaller Fulgoroidea (planthoppers) and other small jumping Auchenorrhyncha (Burrows, 2009a; Burrows and Bräunig, 2010). First, their jumps were propelled by the rapid movements of both hind legs acting in unison. Second, the kinematics revealed that the initial and critical movements during take-off were depression of the hind trochantera powered by large trochanteral depressor muscles in the metathoracic cavity, accompanied after a short delay by extension of the hind tibiae moved by a smaller extensor muscle in each hind femora. Third, the high-speed videos showed that only the hind legs moved in a way that would provide propulsion for a jump. The other legs lost contact with the ground before take-off and showed no pattern of movements that would indicate that they contributed to propulsive forces. Fourth, the wings similarly made no contribution to forward thrust as they remained folded along the body until variable times after take-off, when they would often start to beat as the insect made a smooth transition into flapping flight. The power for a jump must therefore be generated by muscles moving the hind legs, particularly those depressing the hind trochantera. Finally, calculations of the power required of these muscles in the fastest jumps gave values that were 40 times greater than the capabilities of normal muscle (Askew and Marsh, 2002; Ellington, 1985; Josephson, 1993; Weis-Fogh and Alexander, 1977). The implication is therefore that power amplification must be used to enable the trochanteral depressor muscles to generate the jump in a catapult-like action.

Further evidence for a catapult mechanism in these insects came from the observation that the hind legs were always moved into the same position before a jump; both hind trochantera were always fully levated about the coxae. A mechanism must thus be present that allows the depressor muscles to contract without initially moving the legs. Similar requirements are fulfilled in jumps by locusts (Bennet-Clark, 1975; Burrows, 1995; Heitler and Burrows, 1977), in strikes by the raptorial appendages of mantis shrimp (Burrows, 1969; Patek et al., 2007), and in particular by much smaller jumping cercopoid froghoppers (Burrows, 2007b), and many other fulgoroid planthoppers (Burrows and Bräunig, 2010) in the same super family as the lantern bugs. The power-producing muscles (trochanteral depressors) contract in advance of the jump, but the hind legs do not move, allowing a longer time for the necessary force to be built up and energy stored in mechanical distortions of the pleural arches of the metathoracic skeleton. The stored energy is then released rapidly. Without such a catapult mechanism, contractions of the muscles acting directly on the levers of the hind legs would not be able to deliver the power necessary to extend them so rapidly and deliver such high take-off velocities.

In the lantern bugs, the front and middle legs, which were 20–40% shorter than the hind legs, principally because they had much shorter tibiae, had no role in generating thrust during jumping. They did, however, have an important role in setting the elevation and azimuth of jumps by movements and postural adjustments that preceded the rapid propulsive movements of the hind legs. Use of the front and middle pairs of legs in this manner has also been observed in other fulgoroids, including dicytyopharids (Burrows, 2014a) and issids (Burrows, 2009a).

### How do lantern bugs compare with other jumping insects?

**Fig. 7.**
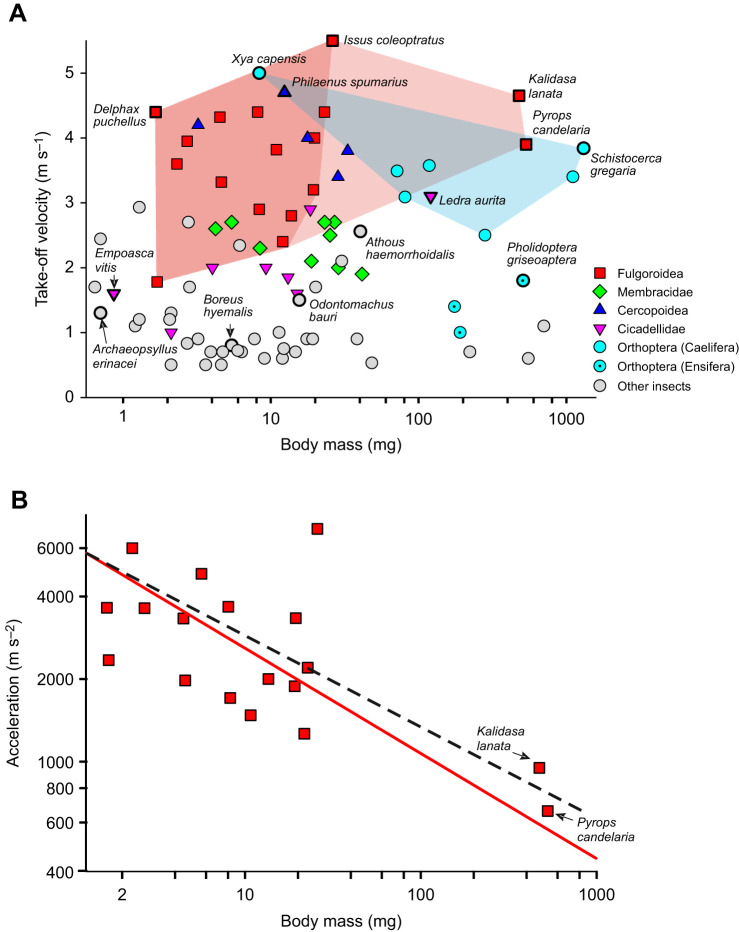
**Jump performance of *K. lanata* and *P. candelaria* relative to other jumping insects.** (A) Distribution of maximum take-off velocities on the basis of body mass of 88 species of jumping insects drawn from the Hemiptera, Orthoptera and other orders. The dark red area shows the extent of body masses and take-off velocities found in planthoppers prior to the characterisation of the much larger lantern bugs described in this paper. The data considerably extend the body mass range over which planthoppers have been demonstrated to perform high-velocity jumps (light red region). The cyan region shows the mass/velocity zone occupied by grasshoppers and allies (Caelifera). The positions of species named in the text are labelled. The lantern bugs have take-off velocities equivalent to those of the fastest of the smaller hemipterans and have similar or faster take-off velocities compared with orthopterans of similar sizes. (B) Plots of the acceleration, leading to take-off, against body mass in *K. lanata* and *P. candelaria* compared with other fulgoroids (Burrows, 2009a,b). The dashed line indicates the expected acceleration based on isometric scaling (slope −0.333); the fitted line (solid) does not differ significantly from this expectation.

Weighing up to 500 mg or more as adults, the lantern bugs (Fulgoridae) studied here are 300 times heavier than the smallest planthoppers within the Fulgoroidea such as *Delphax puchellus* (Delphacidae; M.B., personal observation), 150 times the mass of small froghoppers such as *Philaenus spumarius* (Aphrophoridae; Burrows, 2006), and 600 times heavier than small leafhoppers (Cicadellidae) such as *Empoasca vitis*, which weigh as little as 0.8 mg (Burrows, 2007a; Fig. 7A, Table 3). Prior to this study, the heaviest fulgoroid to have had its jumping performance measured was *I. coleoptratus*, with the fastest take-off velocity of 5.5 m s^−1^, but which at 26 mg was approximately one-twentieth of the mass of the lantern bugs (Burrows, 2009a). More broadly among the Auchenorrhyncha, the heaviest species for which there are data, the cicadellid *Ledra aurita* (M.B., personal observation) is only one-fifth of the mass (93 mg) of the lantern bugs, and its jumping performance is worse, producing a maximum take-off velocity of 3.1 m s^−1^. Even though *K. lanata* and *P. candelaria* do not quite match the performance of *I. coleoptratus*, they remain amongst the fastest of jumping insects, able to perform take-offs in the vicinity of 4 m s^−1^ or above (Fig. 7A). Some cicadas are larger and heavier, but it has yet to be established whether they are able to jump.

**Table 3.**
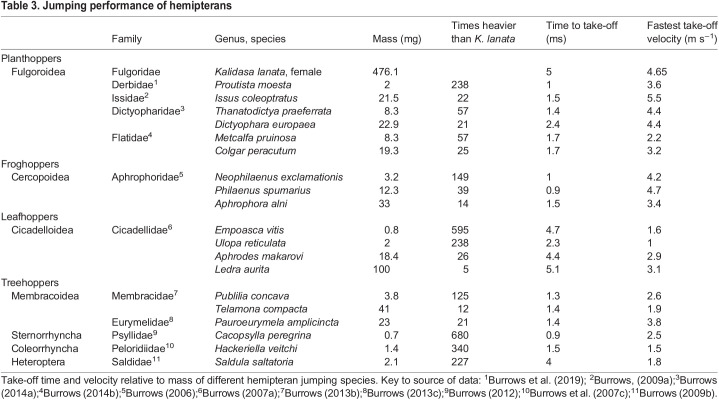
Jumping performance of hemipterans

Jumping is widespread across the insects and has evolved independently many times, which is reflected by the adoption of widely different propulsive devices (grey symbols in Fig. 7A) including: abdominal appendages in springtails (Collembola; Brackenbury and Hunt, 1993; Christian, 1978, 1979), thoracic movements in click beetles (Elateridae, e.g. *Athous haemorrhoidalis*; Evans, 1972, 1973; Kaschek, 1984: Bolmin et al., 2019) and jaw movements in trap-jaw ants (*Odontomachus* sp.; Gronenberg, 1995; Patek et al., 2006). Simply flapping the wings may suffice to propel take-off in some insects, but others need to supplement this with leg movements, as in butterflies (Papilionoidea; Bimbard et al., 2013; Sunada et al., 1993) and dolichopodid flies (Burrows, 2013a). In scorpion flies (Mecoptera), however, clipping the wings does not affect take-off performance driven by leg movements (Burrows, 2019). Different combinations of legs may also be used for propulsion: fruit flies (*Drosophila*) use the middle pair of legs (Card and Dickinson, 2008; von Reyn et al., 2017), and the snow flea *Boreus hyemalis* (Mecoptera: Boreidae) uses simultaneous movement of both the middle and hind legs to propel jumps (Burrows, 2011).

The fastest take-off velocities by jumping insects (>3 m s^−1^) shown in Fig. 7A are dominated by two groups: the Auchenorrhyncha (particularly the Fulgoroidea and Cercopoidea) and the Orthoptera (specifically, the grasshoppers and related groups in the Caelifera) that jump using their hindlegs. Even fleas, considered to be high-performance jumpers, are poor in comparison (e.g. the hedgehog flea *Archaeopsyllus erinacei*; Sutton and Burrows, 2011; Fig. 7A). The Auchenorrhyncha and Caelifera together span over three orders of magnitude in body mass, but prior to the characterisation of jumping performance by the large lantern bugs *K. lanata* and *P. candelaria*, all fast fulgoroids, which use the PADT jumping mechanism, have been small (<30 mg; darker red region in Fig. 7A), and most Caelifera, which jump using the FT mechanism, have been larger (>50 mg; cyan region in Fig. 7A). The addition of these two lantern bugs dramatically extends the range over which the fulgoroids have been demonstrated to operate as high-performance PADT jumpers (light red zone in Fig. 7A) and encompasses the zone previously solely occupied by grasshoppers. Indeed, the take-off velocities achieved by lantern bugs exceed those achieved by even the fastest large grasshoppers, such as solitarious phase *Schistocerca gregaria* (Rogers et al., 2016).

### What effect does the large mass have on jumping?

Larger animals increasingly have the option of not using a catapult mechanism but instead using direct muscle contraction to operate long limbs, which act as levers; this strategy is seen in bush crickets (Tettigonidae/Ensifera) such as *Pholidoptera griseoaptera* (Fig. 7A; Burrows and Morris, 2003). Jumping actuated by direct muscle contraction becomes more important as body size increases above 500–1000 mg (Fig. 7A): a model developed by Sutton et al. (2019) predicts that at body masses >2–3 g, direct muscle actuated jumping will outperform catapult-actuated mechanisms as the inefficiency of converting muscular energy into elastic energy becomes more critical, while the constraints imposed by high muscular strain rates become relatively less important. Only the largest insects approach this boundary.

All our observations, however, have indicated that jumping in lantern bugs is generated by the hind legs in a catapult-like action, and that their performance is amongst the best of jumping insects (Table 3). Energy density is proportional to the square of velocity (energy density=1/2 velocity^2^) and this means that even small differences in take-off velocity can incur large energy costs: the fastest planthopper (the 26 mg *I. coleoptratus*, take-off velocity: 5.5 m s^−1^) has twice the energy density (15.1 J kg^−1^ of body mass) of the locust *S. gregaria* (1300 mg, take-off velocity, 3.84 m s^−1^, energy density 7.37 J kg^−1^). *Pyrops candelaria* and *K. lanata* have energy densities of 7.6 and 11.0 J kg^−1^, respectively, the former resembles that of the locust, whilst the latter approaches the energy densities seen in much smaller fulgoroids, which suggests that larger size does not necessarily incur a substantial decrease in achievable energy densities. The apparent disjunct between small Auchenorrhyncha and larger Orthoptera is likely an accident of sampling; in reality, a substantial overlap in size and performance likely exists.

Greater mass increases the inertia of jumping insects, and with it the possibility of self-inflicted injury through damage to internal tissues or the skeleton of the legs. The physical costs imposed by large size and fast take-off velocities could be mitigated in two ways. First, by increasing the size of the propulsive hind legs to make them sturdier. The data on hind femur length (Fig. 1E,F) indicate that the large lantern bugs are near isometrically scaled-up versions of smaller planthoppers and there was no evidence that the hind femora have been reinforced to resist forces generated during a jump. The second method by which inertia can be decreased is to reduce the acceleration by increasing the time to take-off. The disproportionately long hind tibiae in the large lantern bugs, which show positive allometry (Fig. 1F), are suggestive of a means by which this could be achieved. In a PADT mechanism, however, the ground reaction forces are only a function of the femur length, as the tibiae act as linear struts that control the direction (but not the magnitude) of the ground reaction forces (Burrows and Sutton, 2008). We are left puzzled as to why the tibiae show positive allometry.

Even with isometrically scaled hind femora, acceleration will decrease as a direct consequence of longer leg lengths (Fig. 7B). The measured accelerations of planthoppers are close to the slope of −0.33 that would be expected from isometric scaling, with a fitted regression slope of −0.38 (range −0.26 to −0.56; significance of regression: *R*^2^=0.45, *P=*0.002). This was not significantly different from an isometric relationship (*R*_15_=−0.191, *P*=0.448). As a consequence, the power (*E*/*t* in Table 2) requirements for a jump are less, even if available leverage also increases as leg length increases. The fastest acceleration times by female *K. lanata* (5 ms) were more than five times as long as some froghoppers and some derbid planthoppers (Table 3). The accelerations seen within the planthoppers including *P. candelaria* and *K. lanata* are consistent with their hind femur lengths.

The take-off times in large lantern bugs are likely to be sufficient to challenge the response times of the most alert and agile predators, and compare favourably with the >30 ms acceleration times shown by locusts (Rogers et al., 2016). The conclusion is that large lantern bugs can generate take-off velocities equivalent to their smaller relatives, while being an order of magnitude heavier, without any major morphological or kinematic changes.
